# Unusual Air Crescents on Chest Radiographs

**DOI:** 10.5334/jbsr.3583

**Published:** 2024-06-20

**Authors:** Amanda Marlène Missi, Denis Tack, Nigel Howarth, Hanna Salame

**Affiliations:** 1 Université libre de Bruxelles (Fosfom), Brussels, Belgium; 2 Université libre de Bruxelles, Brussels, Belgium; Department of Radiology, Epicura Hospital, Ath, Belgium; 3 Hirslanden-Clinique des Grangettes, Genève, Switzerland; 4 Department of Radiology, Epicura Hospital, Ath, Belgium

**Keywords:** Air crescent, unusual aetiologies, chest radiograph

## Abstract

The air crescent (AC) is a common radiological sign. Even if its commonest aetiology remains pulmonary aspergillosis, various other causes have been described. In this study, we report four rare causes of ACs seen on chest radiographs that haven’t been described in the literature.

*Teaching point:* The differential diagnosis of an air crescent sign on chest radiographs includes oesophageal bezoar, interstitial lung emphysema, central bronchial stenosis and perforated emphysematous cholecystitis.

## Introduction

The air crescent (AC) is a half-moon collection of gas in the periphery of a cavitary nodule or mass, separating the nodule or mass from the cavity wall [1]. If the commonest reported cause of AC is invasive pulmonary aspergillosis [2], additional identified aetiologies are: pulmonary fungus, lung abscess, textiloma (gossypiboma), lung carcinoma, metastasis, teratoma, Rasmussen aneurysm, Staphylococcal pneumonia or blood clots into a cavity [3, 4]. In this study, four other rare causes of ACs seen on chest radiographs are reported.

## Case Presentations

### Oesophageal bezoar

**Figure 1:**
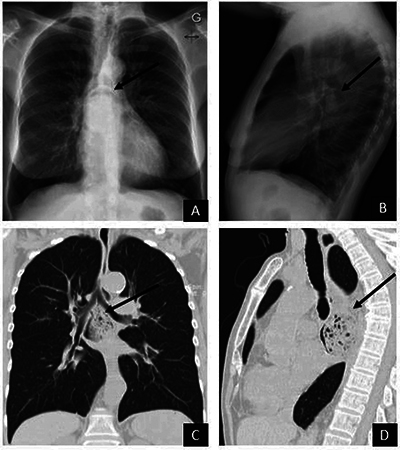
Chest radiography shows an AC in the middle mediastinum in anteroposterior **(A)** and lateral **(B)** views. A CT scan shows a foreign body mass within the thoracic oesophageal lumen **(C)** with an AC located anterior to the mass **(D)**.

A 79-year-old woman was consulted for a persistent cough without infectious signs. A chest X-ray (Figures 1A and 1B) showed a cavity located posteriorly and caudally to the carina, containing a rounded opacity separated from the wall of the cavity by an airspace in a crescent shape. A coronal reformatted chest computed tomography (CT) identified a heterogeneous mass inside the thoracic oesophageal lumen (Figure 1C). Sagittal reformatting (Figure 1D) showed that the AC changed position, now anterior to the mass. The numerous air bubbles within the mass were suggestive of a bezoar, which was confirmed and removed during endoscopy.

### Pulmonary interstitial emphysema

**Figure 2:**
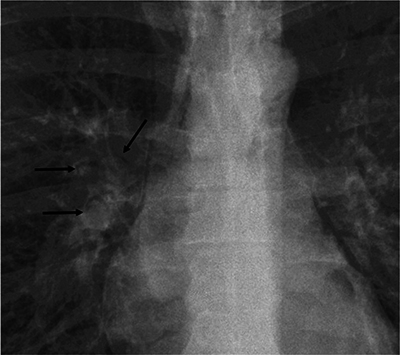
Right hilar air crescent on a chest radiograph (arrows).

A chest X-ray in a 22-year-old asthmatic patient admitted for dyspnoea revealed vertical translucent lines in the mediastinum and right hilar vessels surrounded by AC (Figure 2), indicating a pneumomediastinum with pulmonary interstitial emphysema.

### Bronchial stenosis

**Figure 3:**
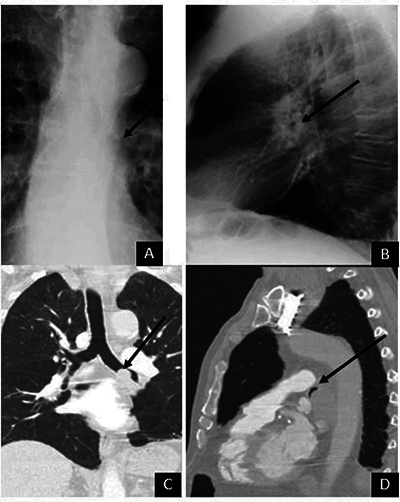
Chest radiography shows an AC (arrows) inside the lumen of the left mainstem bronchus on the lateral view **(B)** and an iceberg sign on the frontal view **(A)**. Coronal **(C)** and sagittal **(D)** reformatted CT showing the bronchial tumour associated with an AC.

A 74-year-old man was admitted for an exacerbation of chronic airway disease. The chest X-ray was reported as normal. On second reading, however, an AC was identified within the lumen of the left mainstem bronchus on lateral view, along with an iceberg sign on frontal view (Figures 3A and 3B), corresponding to bronchial tumoral stenosis. This was confirmed by the chest CT (Figures 3C and 3D) and bronchoscopy.

### Perforated emphysematous cholecystitis

**Figure 4:**
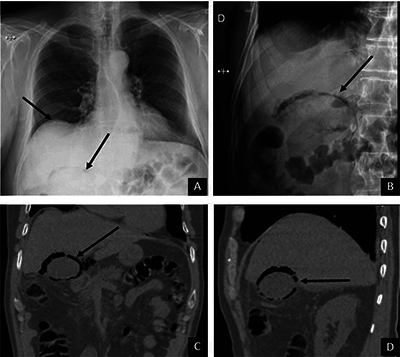
Chest radiography shows a pneumoperitoneum and an AC in the right hypochondrium **(A)**. A supine abdominal X-ray confirmed the AC at the level of the gallbladder bed **(B)**. Unenhanced abdominal CT scan shows air within or next to the gallbladder wall **(C** and **D)** related to emphysematous cholecystitis.

A 67-year-old man consulted at the emergency room with right lateral chest pain. A chest X-ray showed a pneumoperitoneum and an AC in the right upper abdominal quadrant, overlying the liver (Figure 4A) and was confirmed by an abdominal X-ray (Figure 4B). An unenhanced abdominal CT showed air with a crescent shape within the gallbladder wall, leading to the diagnosis of perforated emphysematous cholecystitis (Figures 4C and 4D).

## Comments

The main aetiology of AC seen on chest radiographs remains aspergillus and its complications [4]. Aspergilloma typically manifests as a single, spherical nodule inside a pre-existing cavity, mobile between the supine and prone positions [3]. To date, all reported differential diagnoses of AC involve pathology related to lung parenchyma.

Herein, three differential diagnoses of AC not involving the lung parenchyma are reported: related to the gastro-intestinal tract (oesophageal bezoar), to the gallbladder (perforated emphysematous cholecystitis) and to the tracheobronchial tree (bronchial stenosis).

In the oesophageal bezoar, the AC was located in the mediastinum, and the relative mobility of the mass was suggesting an oesophageal foreign body. In the case of perforated emphysematous cholecystitis, air bubbles resulting from vesicular inflammation were trapped between the liver parenchyma and the gallbladder, thus producing an AC. Careful reading of the radiological images can differentiate bronchial to lung localisation of AC, as described in our third case. The aetiology of the bronchial stenosis was a malignant lesion, narrowing the bronchial lumen into a crescent shape.

In addition to these three extra-parenchymal aetiologies, a case of pulmonary interstitial emphysema as a cause of AC is reported. In this pathology, the lung interstitial tissue is disrupted by the elevated air pressure inside the alveoli and alveolar airspaces, causing air leakage to the interstitium [5].

The range of differential diagnoses of AC is extensive, with mediastinal and infradiaphragmatic pathologies requiring attention when facing an AC on a chest radiograph.
